# Antibiotic Resistance: From the Bench to Patients

**DOI:** 10.3390/antibiotics8030129

**Published:** 2019-08-27

**Authors:** Márió Gajdács, Fernando Albericio

**Affiliations:** 1 Department of Pharmacodynamics and Biopharmacy, Faculty of Pharmacy, University of Szeged, Dóm tér 10., 6720 Szeged, Hungary; 2 School of Chemistry, University of KwaZulu-Natal, Durban 4001, South Africa; 3 Department of Organic Chemistry, University of Barcelona, CIBER-BBN, 08028 Barcelona, Spain

The discovery and subsequent clinical introduction of antibiotics is one of the most important game-changers in the history of medicine [1]. These drugs have saved millions of lives from infections that would previously have been fatal, and later, they allowed for the introduction of surgical interventions, organ transplantation, care of premature infants, and cancer chemotherapy [2]. Nevertheless, the therapy of bacterial infections is becoming less and less straightforward due to the emergence of multidrug resistance (MDR) in these pathogens [3]. Direct consequences of antibiotic resistance include delays in the onset of the appropriate (effective) antimicrobial therapy, the need to use older, more toxic antibiotics (e.g., colistin) with a disadvantageous side-effect profile, longer hospital stays, and an increasing burden on the healthcare infrastructure; overall, a decrease in the quality-of-life (QoL) and an increase in the mortality rate of the affected patients [4,5]. To highlight the severity of the issue, several international declarations have been published to call governments around the globe to take action on antimicrobial resistance [6,7,8,9].

Since the 1980s, pharmaceutical companies have slowly turned away from antimicrobial research and towards the drug therapy of chronic non-communicable diseases [10,11]. New antimicrobials are usually used as last-resort agents in a narrow patient population, resulting in smaller profits [12]. Additionally, drug companies are failing to keep up with the developments in global resistance levels; development of non-susceptibility to the novel antibiotics is inevitable, shortening the period of clinical usefulness of these drugs [13]. During the last years, a number of antibiotics have received marketing authorization from the Food and Drug Administration (FDA) and the European Medicines Agency (EMA) (Table 1) [14,15,16]. 

Although the number of newly marketed antibiotics and the current state of the antimicrobial pipeline offers hope (owing to government-funded research programs and public–private partnerships, generating incentive for pharmaceutical companies), there are several pathogens where providing appropriate therapy is still a major concern [10,11,12,13]. Based on their resistance levels and clinical significance, the so-called “ESKAPE” pathogens (Table 2) receive the utmost attention when it comes to the development of novel antimicrobials [17,18,19,20,21]. This was further highlighted after the World Health Organization declared these microorganisms as priority pathogens for pharmaceutical companies [22]. 

One of the main driving forces behind the development of antibacterial drug resistance is the misuse and overuse of these drugs, both in human medicine and in agriculture [1,2,3]. Thus, programs and interventions aiming at optimizing the use of antimicrobial drugs (such as implementation of policies and guidelines, drug utilization reports, point prevalence surveys, both locally and internationally), collectively termed “antimicrobial stewardship”, have received substantial attention [23]. Antimicrobial stewardship includes decisions like the selection of the dose and duration of the most appropriate antimicrobial(s) for the patient with limited or no side effects, ensuring minimal impact on local resistance levels, ensuring their availability and efficacy for the future [24]. In addition, the implementation of rapid diagnostic techniques in clinical microbiology laboratories (diagnostic stewardship) to aid the choice of drug therapy is another emerging facet of antimicrobial stewardship [25]. This is also highlighted in scientific research; while in 2008, there were only *n* = 45 articles on this topic, in 2018, a nearly twenty-fold increase was observed (*n* = 804). To attain changes clinical practice, the appropriate attitude of healthcare professionals and their continuous professional development is of utmost importance [26,27]. 

Considering the importance of antibiotic resistance and its effects on the QoL of patients and on the state of healthcare infrastructures as a whole, it is our pleasure to co-edit the Special Issue in *Antibiotics*, termed “Antibiotic Resistance: From the Bench to Patients”. The Special Issue contains excellent quality research articles and comprehensive review papers on the epidemiology of various MDR pathogens worldwide, novel diagnostic and point-of-care (POCT) tests, interventional studies on antimicrobial drug utilization and pharmaco-epidemiological studies. In addition, the Special Issue welcomes reports on the knowledge, attitude, and practice of healthcare professionals (nurses, doctors, pharmacists, etc.) and patients regarding antibiotics and antibiotic resistance.

## Figures and Tables

**Table 1:** Antibiotics recently approved by the Food and Drug Administration (FDA) and/or European Medicines Agency (EMA).

Active Pharmaceutical Ingredient	Trade Name	Class/Comments
Doripenem	Doribax ^™^ (US) Finibax ^™^ (EU)	Carbapenem
Ceftaroline fosamil	Teflaro ^™^ (US) Zinforo^™^ (EU)	Cephalosporin
Ceftobiprole medocaril	Zevtera^™^(US) Mabelio^™^ (EU)	Cephalosporin
Ceftolozane/tazobactam	Zerbaxa ^™^ (US/EU)	Combination antibiotic: cephalosporin/β-lactamase inhibitor
Ceftazidime/avibactam	Avycaz^™^ (US/EU)	Combination antibiotic: cephalosporin/β-lactamase inhibitor
Meropenem/vaborbactam	Vabomere^™^ (US) Carbavance^™^ (EU)	Combination antibiotic: carbapenem/β-lactamase inhibitor
Imipenem/cilastatin/relebactam	Recarbrio ^™^ (US/EU)	Combination antibiotic: carbapenem/renal dehydropeptidase inhibitor/β-lactamase inhibitor
Telavancin	Vibativ^™^ (US)	Derivatives of either vancomycin or lipoglycopeptide
Dalbavancin	Dalvance^™^ (US) Xydalba^™^ (EU)	Derivatives of either vancomycin or lipoglycopeptide
Oritavancin	Orbactiv^™^ (US/EU)	Derivatives of either vancomycin or lipoglycopeptide
Eravacycline	Xerava^™^ (US/EU)	Tetracycline derivatives
Sarecycline	Seysara^™^ (US)	Tetracycline derivatives
Omadacycline	Nuzyra^™^ (US)	Tetracycline derivatives
Bedaquiline,	Sirturo^™^ (US/EU)	Diarylquinoline (DARQ)
Tedizolid	Sivextro ^™^ (US/EU)	Oxazolidinone
Delafloxacin meglumine	Baxdela^™^ (US)	Fluoroquinolone
Plazomicin	Zemdri ^™^ (US)	Next-generation aminoglycoside (neoglycoside)
Lefamulin	Xenleta^™^ (US)	Pleuromutilin

US: Trade name in the United States; EU: trade name in the member states of the European Union.

**Table 2:** Current list of ESKAPE pathogens.

Pathogens
*Enterococcus faecium*
*Staphylococcus aureus* *(Stenotrophomonas maltophilia)*
*Klebsiella pneumoniae* *(Clostridioides difficile)*
*Acinetobacter* spp.
*Pseudomonas aeruginosa*
*Enterobacter* spp. (members of *Enterobacterales*)
